# In Vitro Evaluation of the Antiviral Activity of Polyphenol (-)-Epigallocatechin-3-Gallate (EGCG) Against Mayaro Virus

**DOI:** 10.3390/v17020258

**Published:** 2025-02-14

**Authors:** Pâmela Jóyce Previdelli da Conceição, Gabriela Miranda Ayusso, Tamara Carvalho, Maria Leticia Duarte Lima, Mikaela dos Santos Marinho, Fábio Rogério Moraes, Paola Elaine Galán-Jurado, José González-Santamaría, Cíntia Bittar, Bo Zhang, Ana Carolina Gomes Jardim, Paula Rahal, Marilia Freitas Calmon

**Affiliations:** 1 Institute of Biosciences, Letters and Exact Sciences, São Paulo State University, São José do Rio Preto 15054-000, SP, Brazil; pamela.joyce@unesp.br (P.J.P.d.C.); gabriela.ayusso@unesp.br (G.M.A.); maria-leticia.lima@unesp.br (M.L.D.L.); p.rahal@unesp.br (P.R.); 2 Institut de Recherche en Infectiologie de Montpellier, Centre National de la Recherche Scientifique (CNRS), 34000 Montpellier, France; tamara@wcarvalho.com; 3 Laboratory of Antiviral Research, Institute of Biomedical Science, ICBIM/UFU, Uberlândia 38405-302, MG, Brazil; mikamarinho@yahoo.com.br (M.d.S.M.); jardim@ufu.br (A.C.G.J.); 4 Physics Department, São Paulo State University—UNESP, São José do Rio Preto 15385-000, SP, Brazil; fabio.moraes@unesp.br; 5 Grupo de Biología Celular y Molecular de Arbovirus, Departamento de Genómica y Proteómica, Instituto Conmemorativo Gorgas de Estudios de la Salud, Panamá City 0816-02593, Panama; paola.elaine.jurado@gmail.com (P.E.G.-J.); jgonzalezsantamaria@gorgas.gob.pa (J.G.-S.); 6 Laboratory of Molecular Immunology, The Rockefeller University, New York, NY 10065, USA; cibittar@gmail.com; 7 Laboratory of Virology and Biosafety, Wuhan Institute of Virology, Chinese Academy of Sciences, Wuhan 430071, China; zhangbo@wh.iov.cn

**Keywords:** Mayaro, antivirals, EGCG

## Abstract

The Mayaro virus (MAYV), *Togaviridae* family, genus *Alphavirus*, has caused several sporadic outbreaks, affecting countries in the Americas. Currently, there are no licensed drugs against MAYV, requiring the search for effective antiviral compounds. Thus, this study aimed to evaluate the antiviral potential of polyphenol (-)-epigallocatechin-3-gallate (EGCG) against MAYV infection, in vitro. Antiviral assays against MAYV were performed in BHK-21 and Vero E6 cells. In addition, molecular docking was performed with EGCG and the MAYV non-structural and structural proteins. EGCG showed a significant protective effect against MAYV infection in both cell lines. The virucidal assay showed an effect on extracellular viral particles at the entry stage into BHK-21 cells. Finally, it also showed significant inhibition in the post-entry stages of the MAYV replication cycle, acting on the replication of the genetic material and late stages, such as assembly and release. In addition, the MAYV proteins E1 and nsP1 were significantly inhibited by the EGCG treatment in BHK-21 cells. Molecular docking analysis also showed that EGCG could interact with MAYV Capsid and Envelope proteins (E1 and E2). Therefore, this study shows the potential of EGCG as a promising antiviral against MAYV, as it acts on different stages of the MAYV replication cycle.

## 1. Introduction

Arboviruses (*Arthropod-borne viruses*) are a group of approximately 500 species of viruses distributed in 14 different families. They have as their main feature the transmission between vertebrate hosts and hematophagous arthropod vectors, such as mosquitoes and ticks [1,2]. The Mayaro virus (MAYV), belonging to the *Togaviridae* family, genus *Alphavirus*, is an enveloped virus and has a positive-sense, single-stranded RNA genome of 11.5 kb. MAYV was isolated for the first time in Trinidad and Tobago in 1954 [3,4]. Since then, MAYV has caused several sporadic outbreaks, affecting mainly rural areas and forest regions of the Americas [5,6].

Brazil has the highest number of confirmed cases of Mayaro in the world, with the highest exposure rate in the northern, being transmitted by the *Haemagogus* mosquito. However, studies have demonstrated the possibility of MAYV transmission by urban vectors, such as *Aedes aegypti* [7,8]. Epidemiological studies have also shown an increase in the number of infections caused by MAYV in big cities and previously unaffected areas [9]. Altogether, these data together are strong indicators of the silent urbanization of MAYV in Brazil [10,11]. Furthermore, Brazil has a great diversity of arthropods and vertebrate animals, which, in combination with climatic conditions, constitute favorable characteristics for the occurrence of these diseases [1].

To date, few asymptomatic cases of MAYV infection have been reported [12]. The symptomatic disease is typically characterized by high fever, headache, myalgia, arthralgia, and joint swelling [10,12,13,14]. It is also known that MAYV can cause serious complications, which can progress to chronic arthritis, and cause neurological complications, myocarditis, and hemorrhagic manifestations [13,15,16,17].

The search for new antiviral substances has intensified in the last decade due to the limited number of drugs in parallel with the high rate of antiviral resistance and the increasing number of infections caused by emerging and reemerging viruses [18,19]. As a result, many natural compounds have been explored in several studies, searching for therapeutic properties [18,20,21]. Teas made from *Camellia sinensis* leaves are of great interest, as they have been produced and used for years, bringing many health benefits. It shows antioxidant, neuroprotective, cardioprotective, anticancer, antiobesity, anti-inflammatory, and antimicrobial effects. Therefore, it is of great interest to obtain bioactive compounds from these plant species [22,23,24].

The (-)-epigalocatechin-3-gallate (EGCG), which is one of the main polyphenolics catechins present in green tea presented an intense antiviral activity against several viruses. Studies have shown the activity of EGCG against human immunodeficiency virus (HIV) [25], influenza virus (FLU) [26,27], herpes simplex virus (HSV) [28], hepatitis C virus (HCV) [29], and SARS-CoV-2 [30,31]. EGCG also has an effect against several arboviruses, including chikungunya (CHIKV), which belongs to the same genus as the MAYV, *Alphavirus* [32,33]. The flaviviruses Zika virus (ZIKV), West Nile (WNV), and dengue virus (DENV) have also been the subject of studies involving EGCG [34,35]. These studies showed that EGCG was able to inhibit the entry and post-entry stages of viral infection in host cells. Furthermore, EGCG also acted directly on viral particles [32,33,35].

Therefore, this study investigates the action of EGCG as an antiviral against MAYV, which is a neglected and little-studied arbovirus to date.

## 2. Materials and Methods

### 2.1. (-)-Epigallocatechin Gallate (EGCG)

**Figure 1:**
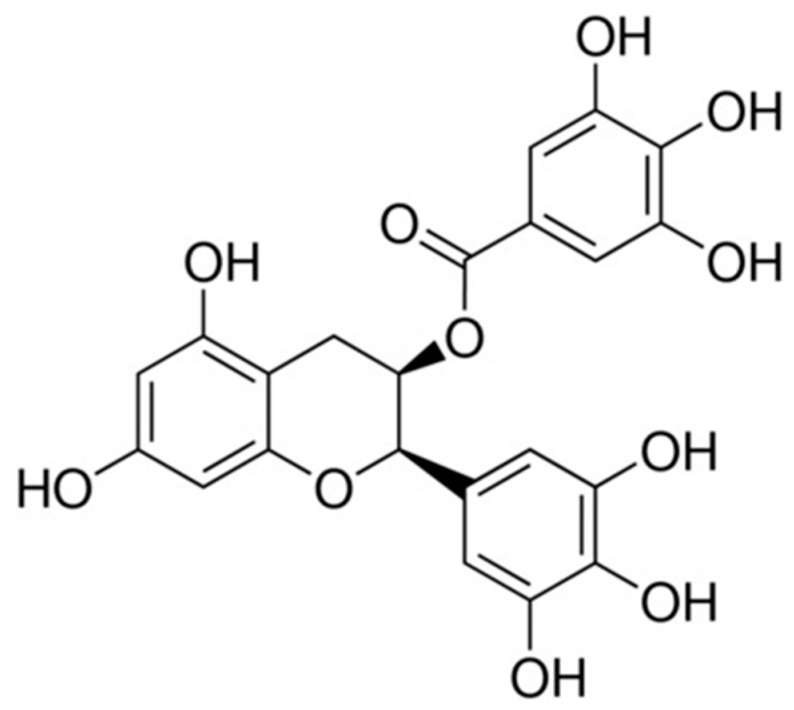
Structural formula of (-)-Epigallocatechin gallate (EGCG).

EGCG (Sigma-Aldrich, Missouri, USA) was dissolved in DMSO to prepare a stock solution (i.e., of 5 mg/mL EGCG), which was then stored at −20 °C until use (Figure 1).

### 2.2. Cells

Baby hamster kidney fibroblast cells (BHK-21) (ATCC CCL10) and African green monkey kidney cells (Vero E6) (ATCC CRL1586) were maintained in a humidified incubator at 37 °C in 5% CO_2_. Both cell lines were cultured in DMEM medium (Dulbecco’s Modified Eagle’s Medium) (Cultilab, São Paulo, Brazil) supplemented with 10% fetal bovine serum (FBS) (Cultilab, São Paulo, Brazil), 1% penicillin (10,000 IU/mL)/streptomycin (10 mg/mL) (Cultilab, São Paulo, Brazil), and 1% amphotericin B (Cultilab, São Paulo, Brazil).

### 2.3. Virus

The MAYV (AVR 0565 strain, San Martin, Peru) provided by Dr. Scott Weaver (WRCEVA, University of Texas Medical Branch, Galveston, TX, USA) was propagated and titrated in BHK-21 cells. We also used an infectious reporter virus, based on the MAYV BeAr20290 strain containing the Nanoluciferase (NLuc) reporter gene, designated as MAYV-*nanoluc*, which was constructed using the cytomegalovirus (CMV) promoter (plasmid CMV-MAYV-*nanoluc*). After transfection into mammal cells, the CMV promoter is directly transcribed into mRNA, which generates MAYV-*nanoluc* virus particles [36].

Initially, we performed amplification and purification of the plasmid CMV-MAYV-*nanoluc*. BHK-21 cells were seeded (1 × 10^5^ cells/well) in 24-well plates for 24 h. Afterwards, transfection was performed with 500 ng of plasmid CMV-MAYV-*nanoluc*, using Lipofectamine 2000 (Thermo Fisher Scientific, Waltham, MA, USA) and OPTI-MEM (Reduced Serum Medium) (Gibco, Waltham, MA, USA) following manufacturer’s instructions. The cells were then incubated for 72 h at 37 °C, 5% CO_2_, until the cytopathic effect in most of the cells for collection, filtration, and storage of the supernatant at −80 °C [36].

For titration, Vero E6 cells were seeded in 24-well plates (1 × 10^5^ cells/well) and incubated at 37 °C, 5% CO_2_, for 24 h until confluence. Then, cells were infected with ten-fold serial dilutions of CMV-MAYV-*nanoluc* infectious clones. Cells were incubated with viruses for 1 h at 37 °C and 5% CO_2_, followed by the inoculum removal, washing with PBS to remove the unbound virus, and the addition of fresh medium supplemented with 1% dilution of stock of penicillin and streptomycin, 2% FBS, and 1.5% carboxymethyl cellulose (CMC) (Sigma Aldrich, St Louis, MO, USA). After 48 h post-infection (h.p.i.) cells were fixed with 10% formaldehyde solution for 20 min and stained with 1% violet crystal for 5 min. We counted the lysis plates and viral titers were expressed in plaque-forming units per mL (PFU/mL).

### 2.4. Cytotoxicity Assay

Cytotoxicity assay was performed using MTT assay [(3-(4,5-dimethylthiazol-2-yl)-2,5-diphenyltetrazolium Bromide) (Sigma Aldrich, Missouri, USA) for determined cell viability. For this assay, cells were seeded in 96-well plates (5 × 10^3^ cells/well) and incubated at 37 °C, 5% CO_2_, for 24 h. Then, the medium was removed and 100 µL of DMEM without amphotericin B containing different dilutions of EGCG (6.75, 12.5, 25, 50, 100, and 200 µg/mL) were added. The EGCG effect on cells was determined at 24 h post-addition of the compound. After this time, the supernatant was removed, 100 µL of MTT solution (1 µg/mL) was added into cells and the plaque was incubated at 37 °C for 30 min. Afterwards, the MTT solution was replaced with 100 µL of dimethyl sulfoxide (DMSO) to solubilize the formazan crystals, and the absorbance was measured at 572 nm using a FLUOstar Omega plate reader (BMG LABTECH, Offenburg, Germany). The cytotoxic concentration of 50% (CC_50_) was calculated using Graph Pad Prism v. 5.

### 2.5. Antiviral Screening Assay

Cells were seeded (1 × 10^4^ cells/well) in 96-well plates and incubated at 37 °C, 5% CO_2_, for 24 h until 70% of confluence. For screening, cells were incubated with EGCG at the maximum non-toxic concentrations (MNTC) together with the CMV-MAYV-*nanoluc* at an MOI of 0.05 for 24 h. Then, the supernatant was removed, and 30 μL of lysis buffer from the Renilla Luciferase Assay Lysis Buffer kit (Promega, Madison, WI, USA) was added to the cells and incubated for 30 min for cell lysis. After, 10 μL of the cell lysate was transferred to a white flat-bottom microplate (Greiner Bio-one, Americana-SP, Brazil), and 50 μL of the substrate was added, coming from the Renilla Luciferase kit, properly prepared. Immediately after adding the substrate, the expression of the Renilla Luciferase protein was detected by reading the luminescence on the FLUOstar Omega plate reader (BMG LABTECH, Baden-Württemberg, Germany).

### 2.6. EGCG Antiviral Dose–Response Assay

Cells were seeded (1 × 10^4^ cells/well) in 96-well plates and incubated at 37 °C, 5% CO_2_, for 24 h until 70% of confluence. Different concentrations of EGCG (6.75, 12.5, 25, 50, 100, and 200 µg/mL) were added to the cells simultaneously with the MAYV-*nanoluc* (MOI 0.05) and incubated for 24 h, at 37 °C, 5% CO_2_. After 24 h, the luminescence reading was performed on the plate reader, as previously described in item 2.5. The obtained EC50 values were calculated using non-linear regression. The values of CC_50_ and EC_50_ were used to calculate the selectivity index (SI = CC_50_/EC_50_).

### 2.7. Evaluation of the Virucidal Effect of EGCG Against MAYV

This assay evaluates the action of the compound on viral particles. Cells were seeded (1 × 10^4^ cells/well) in 96-well plates and incubated at 37 °C, 5% CO_2_, for 24 h until 70% of confluence. The MAYV-*nanoluc* (MOI 5) was incubated with EGCG at MNTC for 1 h, at 37 °C. After, the mixture (EGCG + MAYV) was added to cells for 1 h, at 37 °C for viral adsorption. Then, the supernatant was removed, and cells were washed with PBS, and DMEM medium, with 2% FBS was added. Luminescence levels were measured at 24 h.p.i. to analyze the virus replication rates, as explained earlier.

### 2.8. Time-of-Addition Assay

This assay was carried out using different conditions to evaluate the action of EGCG on host cells, as well as the inhibitory action during the MAYV replication cycle, in stages such as entry and post-entry. For all tests, cells were seeded (1 × 10^4^ cells/well) on 96-well plates and incubated at 37 °C, 5% CO_2_, for 24 h until 70% of confluence. The EGCG was used in MNTC, and the MAYV-*nanoluc* (MOI 0.05). So, BHK-21 and Vero E6 cells were treated with EGCG either pre-, during-, or post-infection. For the pre-treatment, EGCG at 25 μg/mL was added 2 h before infection. At the time of infection, cells were washed three times before the addition of the virus inoculum. For the during-infection condition, EGCG was added together with the MAYV-*nanoluc* at MOI 0.05 and was present for only 1 h. For the post-entry condition, EGCG was added at 1, 2, 4, 6, and 8 hpi. In all experiments, the virus inoculum was removed after one hour, the cells were washed twice with PBS and the medium (pre-infection and during-infection conditions) or the EGCG with medium (post-entry conditions) was added, and the luminescence levels were measured at 24 h.p.i. to analyze the virus replication rates.

### 2.9. Western Blotting

BHK-21 cells were infected with MAYV and after 1 h of virus adsorption, cells were treated with EGCG at 25 μg/mL concentration. Following 24 h of infection, protein extracts were obtained and separated in SDS-PAGE, transferred to nitrocellulose membranes, and blocked with a solution of 5% non-fat milk in T-TBS buffer. Subsequently, membranes were incubated overnight at 4 °C with the following primary antibodies: rabbit polyclonal anti-E1, rabbit polyclonal anti-nsP1 (previously validated) [37], and mouse monoclonal anti-β-actin (Cat # VMA00048, Bio-Rad, Hercules, CA, USA). Afterward, the membranes were washed 3 times with T-TBS buffer and incubated with HRP-conjugated goat anti-rabbit (Cat. # 926-80011) or goat anti-mouse (Cat. # 926-80010) secondary antibodies (LI-COR, Lincoln, NE, USA) for 1 h at room temperature. Finally, the membranes were incubated with SignalFireTM ECL Reagent (Cell Signaling Technology, Danvers, MA, USA) for 5 min, and the chemiluminescent signal was detected with a C-Digit scanner (LI-COR, Lincoln, NE, USA).

### 2.10. Molecular Docking

In order to obtain insights into the structural properties between the interaction of MAYV proteins and EGCG, bioinformatics tools were used. Protein–ligand docking is a widely known technique within the realm of bioinformatics, as it plays a pivotal role in deciphering the intricate interactions between proteins and small-molecule ligands [38]. Although binding energies are known to not be in line with experimental determinations, there is evidence that protein–ligand docking may resemble structural binding modes found in crystalline complexes [39].

For the present study, molecular docking was performed using AutoDock Vina implementation, as available in SeamDock [40], with a box centered in the protein target (Cap, E1 and E2). In total, 100 binding modes were retrieved with an exhaustiveness parameter of 20. The 20 best-ranked poses were used for further analysis. LigPlot+ [41] for ligand binding evaluation and the PyMOL Molecular Graphics System, Version 2.4.0, (Schrödinger, LLC, New York, NY, USA) for structures visualization were used.

#### 2.10.1. Protein Electrostatic Potential Surface

Electrostatics is one of the most important forces driving protein interaction with other molecules [42]. The local charge distribution gives rise to an electrostatic potential surface that is important to stabilize protein–substrate, protein–ligand, protein–protein [42], and protein–support interactions [43]. To evaluate the electrostatic potential pattern on the MAYV Cap, E1, and E2 proteins surface, it was used the PDB2PQR server 1.8 to assign atomic charges and radius based on the AMBER force field [44]. Following this, the Poisson–Boltzmann equation was solved by using the software APBS (Adaptive Poisson–Boltzmann Solver, version 1.3) [45] in neutral water solvent. The parameters available on the APBS webserver were used by default, and 0.15 M of positive and negative charges (radius 2 Å) were set for calculation. The dielectric constants were set to 2.00 and 78.54 for protein and water, respectively. The probe radius for surface assessment was set to 1.4 Å and the temperature 298.15 K was used. PyMol Version 1.5.0.4 (Schrödinger, LLC.) was used for visualization and figure preparation.

#### 2.10.2. Virtual Alanine Scanning

In order to assess individual amino acid residue importance for native structures, the utilization of virtual alanine scanning was explored, using the Robetta web server framework [46]. Virtual alanine scanning is a computational technique involving the systematic substitution of amino acid residues with alanine. By evaluating the binding energy difference for individual residues, it estimated their importance to protein stability and function. The most important residues at interfaces established among proteins, Cap, E1, and E2 were used to assess putative EGCG binding sites.

### 2.11. Statistical Analysis

Results were evaluated using the paired Student’s *t*-test for parametric data and the Mann–Whitney test for non-parametric data, with a *p*-value < 0.05 considered statistically significant. To calculate CC50 and EC50, a non-linear regression analysis of dose–response curves were used. All results were evaluated using Graph Pad Prism software (Graph Pad Software, version 8.0.1, GraphPad Software, Boston, MA USA) and Microsoft Excel (Microsoft, Redmond, WA, USA). All the experiments were performed in three independent experiments. The antiviral analysis was performed in quadruplicate while cytotoxicity analyses were in triplicate.

## 3. Results

### 3.1. Effect of ECGC on Cell Viability

To evaluate the antiviral effect of EGCG and its ability to inhibit MAYV-*nanoluc*, a cytotoxicity assay was first carried out. BHK-21 and Vero E6 cells were treated with different concentrations of EGCG. The MNTC of EGCG was defined as the highest concentration of the compound capable of keeping a minimum of 80% of the cells viable. The results demonstrated that EGCG presented an MNTC of 50 μg/mL in BHK-21 cells, while in Vero E6, it presented an MNTC of 25 μg/mL. We established the use of EGCG at a concentration of 25 μg/mL to carry out antiviral assays involving both cells (Figure S1).

### 3.2. EGCG Shows Antiviral Activity Against MAYV

**Figure 2:**
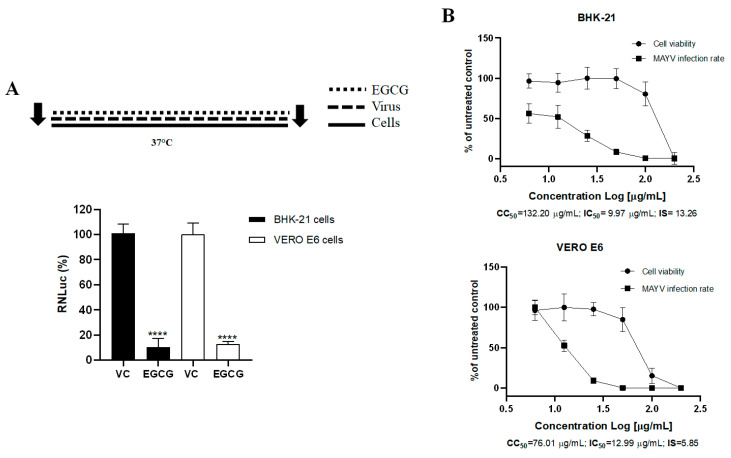
Inhibitory effect of EGCG on MAYV—Nanoluc replication cycle. (**A**) The cells were treated with the EGCG (25 µg/mL) simultaneous with MAYV-*nanoluc* (MOI 0.05) for 24 h with subsequent measurement of RNLuc activity. (**B**) The cells were treated with ECGC at different concentrations (200, 100, 50, 25, 12.5, and 6.25 µg/mL) simultaneously with MAYV-*nanoluc* (MOI 0.05) for 24 h. The percentage of viral replication was measured by luminescence reading and the percentage of cell viability by the MTT assay. 50% cytotoxic concentration (CC50), 50% viral inhibitory effect concentration (EC50), and selectivity index (SI). DMSO 1% was used as vehicle control (VC). Student’s *t* test was used, with data considered significant at ****: *p* < 0.0001.

The results in the initial screening showed that EGCG at 25 μg/mL presented high rates of viral inhibition in BHK-21 (90.5% viral inhibition) and Vero E6 (87.3% viral inhibition) cells (*p* ≤ 0.001), when compared to the vehicle control (VC) (Figure 2).

After carrying out the screening test for the compounds and since ECGC showed activity against the MAYV-*nanoluc*, the dose-dependence test was performed to determine the CC50, EC50, and SI values. Different concentrations were evaluated concomitantly with the assessment of cell viability at these different concentrations. EGCG impaired MAYV-*nanoluc* replication in a dose-dependent manner, reducing the percentage of viral replication with an increase in EGCG concentration until reaching MNTC. The lowest concentration with cell viability above 80% in BHK-21 cells (6.25 µg/mL) obtained a viral replication inhibition rate of 43.8%. For Vero cells, the lowest concentration with cell viability above 80% (25 μg/mL) obtained a replication inhibition percentage of 86.8%. The values of CC_50_, EC_50_, and SI are shown in Figure 2.

### 3.3. Virucidal Effect of the EGCG on MAYV Extracellular Particles

**Figure 3:**
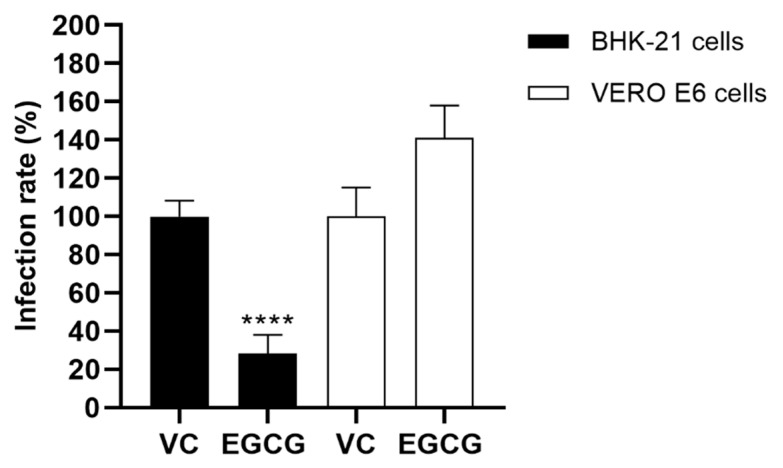
The viral particles were incubated with EGCG (25 μg/mL) for 1 h, after incubation with the cells for 1 h and, after washed with PBS, with subsequent incubation with fresh medium for 24 h. MAYV-*nanoluc* replication was analyzed via the measurement of NLuc activity 24 h.p.i. DMSO 1% was used as vehicle control (VC). Student’s *t* test was used, with data considered significant at; ****: *p* < 0.0001.

The EGCG showed significant virucidal activity on MAYV-*nanoluc* particles in BHK-21 cells compared to vehicle control (71.7%; *p* < 0.0001). On the other hand, EGCG was unable to inhibit MAYV-*nanoluc* replication in Vero E6 cells (Figure 3).

### 3.4. EGCG Shows Prophylactic Effect and Inhibits Early and Late Steps of the MAYV Replication Cycle

**Figure 4:**
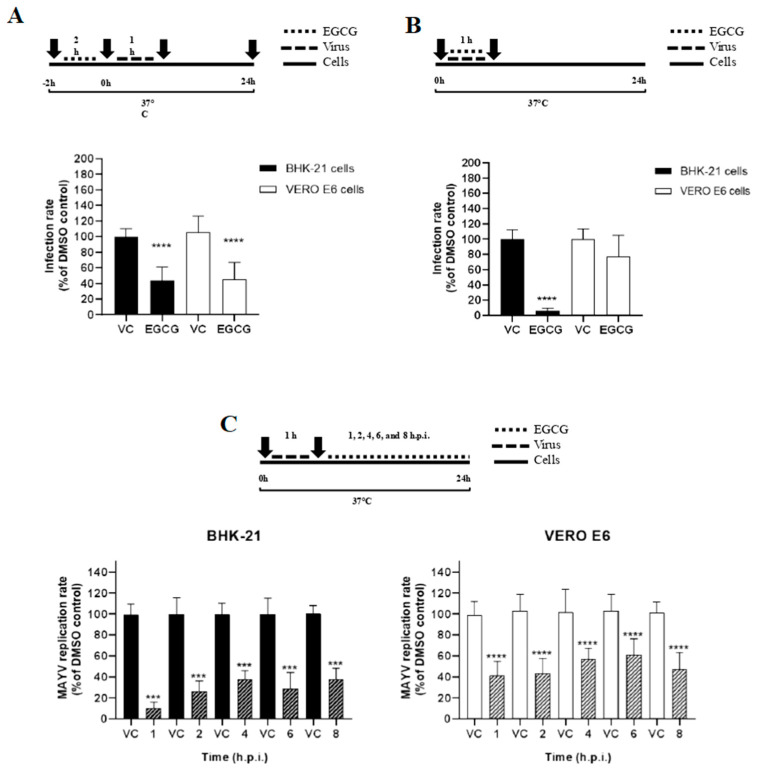
Effect of EGCG on pre-treatment, entry, and post-entry stages of the MAYV-*nanoluc* replication cycle. (**A**) BHK-21 and Vero E6 cells were pretreated with EGCG (25 μg/mL) and, after 2 h, washed with PBS and infected for 1 h (MOI of 0.05), washed with PBS again, with incubation for 24 h and, with subsequent reading of the luminescence. (**B**) The cells were treated with EGCG (25 μg/mL) simultaneous with MAYV-*nanoluc* (MOI of 0.05) for 1 h and, after washed with PBS, with subsequent incubation with fresh medium for 24 h. After 24 h, we read the luminescence. (**C**) The cells were infected with the virus (MOI 0.05) for 1 h; after infection, they were washed with PBS and then treated with EGCG (25 μg/mL) for the indicated times (1,2,4,6 and 8 h.p.i.). MAYV-*nanoluc* replication was analyzed via the measurement of NLuc activity 24 h.p.i. DMSO 1% was used as vehicle control (VC). Student’s *t* test was used, with data considered significant at ***: *p* < 0.001; ****: *p* < 0.0001.

To explore the potential inhibitory mechanisms, a time-of-addition assay of EGCG against MAYV-*nanoluc* was conducted to study the target stage of EGCG during the viral replication cycle. The results showed that the EGCG exhibited the strongest inhibitory in BHK-21 cells in the protective effect (−2 h: pre-treatment) (56.6%; *p* < 0.0001). It was also possible to notice a protective effect, with a 60.7% (*p* < 0.0001) reduction in viral replication in Vero E6 cells (Figure 4A). During infection (0–1 h), the EGCG inhibited 94% of the significant MAYV-*nanoluc* entry stage in BHK-21 cells compared to vehicle control (*p* < 0.0001). However, for Vero E6 cells, no inhibitory effect was observed (Figure 4B). In the post-entry stages of the MAYV-*nanoluc* replication cycle, there was an inhibition percentage of 85.5% (*p* < 0.0001) in BHK-21 cells and 57.7% (*p* < 0.0001) in Vero E6 cells treated with EGCG 1 h.p.i. The treatment with EGCG two h.p.i. presented an inhibition of 73.2% (*p* < 0.0001) in BHK-21 cells and 59.7% (*p* < 0.0001) in Vero E6 cells. In BHK-21 cells treated with EGCG 4, 6, and 8 h.p.i., the percentage of MAYV-*nanoluc* inhibition was 62.4% (*p* < 0.0001), 71.1% (*p* < 0.0001), and 57.5%, respectively, compared to the vehicle control. For hVero E6 cells, the percentage of inhibition in the replication cycle of MAYV-*nanoluc* was 44.9% (*p* < 0.0001), 41.8% (*p* < 0.0001), and 53.8% (*p* < 0.0001) for the times 4, 6, and 8 h.p.i., respectively, showing possible inhibition of late stages in the replication cycle of MAYV-*nanoluc* (Figure 4C).

### 3.5. EGCG Treatment Reduce MAYV E1 and nsP1 Protein Expressions

**Figure 5:**
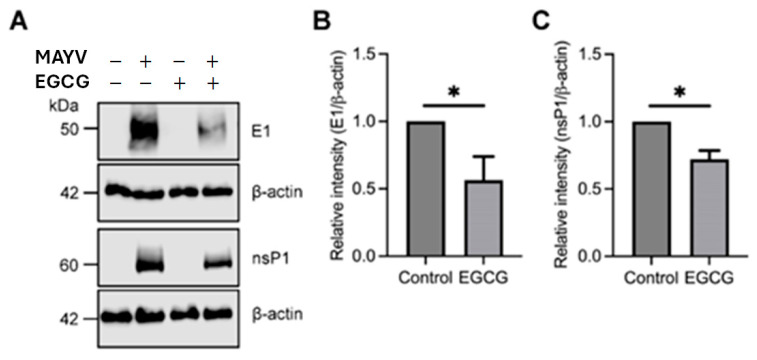
EGCG downmodulates the expression of MAYV E1 and nsP1 proteins. (**A**) Bands of specific proteins can be observed. (**B**) There was a clear reduction in MAYV E1 and (**C**) nsP1 proteins after EGCG treatments for 24 h using Western blot analysis. β-actin protein was used as a loading control. kDa: kilodaltons; WB: Western blot. * *p* ≤ 0.05.

To evaluate the effect of EGCG on viral protein expression, we infected BHK-21 cells with MAYV (AVR 0565 strain) at an MOI of 1 and then the cells were treated with EGCG at 25 μg/mL concentration. After 24 h of incubation, we analyzed the levels of MAYV E1 and nsP1 proteins. As shown in Figure 5, EGCG treatment promoted a clear reduction in the levels of both viral proteins, suggesting that this compound affects the expression of MAYV E1 and nsP1 proteins (Figure 5).

### 3.6. Molecular Docking Analysis Showed Potential Protein-MAYV Targets for the EGCG

**Figure 6:**
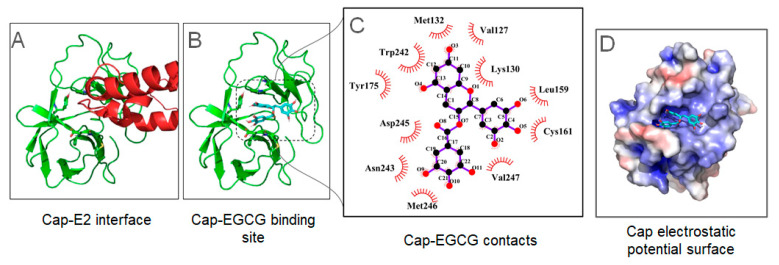
Capsid protein from MAYV docked with EGCG. (**A**) Cap-E2 interaction in crystallographic structure matches the (**B**) EGCG binding site evaluated from docking performed using SeamDock. (**C**) LigPlot+ reveals that EGCG can perform hydrogen bonds with Lys130, Asp245, and Met246. In addition, π-stacking interaction with Tyr175 is important for establishing interaction. Several other hydrophobic interactions are also observed. (**D**) Cap electrostatic potential surface shows a positive binding site for EGCG.

Following the aforementioned methodology, EGCG docked with the capsid protein from MAYV, retrieved from the Protein Data Bank (PDB, [47]) under the code 7KO8, chain A [3]. The ligand EGCG structure was also found in a different PDB, under the code 3NG5, and converted to mol2 file using PyMOL. The 20 best-ranked poses from SeamDock were seen to form three major clusters. The cluster with the greatest number of poses contained 15 out of the 20 best-ranked poses. The proposed EGCG binding site matches the interface between Cap and E2 protein (Figure 5A). Using the lowest energy pose from this binding site, SeamDock lists hydrogen bond formation between EGCG and Cap in residues Lys130, Asp245, and Met246. Also, π-stacking interaction with Tyr175 is observed. Using Pymol to detect polar contacts between Cap and E2, it is found that Lys130, Tyr175, and Asp245 establish contacts to protein E2. Thus, the described binding site is potentially important for drugs to impair virus assembly. Other hydrophobic interactions are observed, with residues Val127, Met132, Trp242, Asn243, Val247, Cys161, and Leu159. Protein electrostatic calculations show EGCG binding site as mainly positively charged (Figure 6D). Virtual alanine scanning has also highlighted the importance of the residues Lys130 and Tyr175 in binding with E2 protein. When these residues are mutated to alanine, a predicted increase in binding energy is estimated to be 0.66 and 1.32 kcal/mol, respectively.

The same approach applied to the E1 protein has revealed that EGCG is able to interact with this protein in several binding modes. Both E1 and E2 proteins form the spike in MAYV, and they are organized as trimers of the heterodimers of E1 and E2. The two best-ranking poses are shown in Figure 6, showing that EGCG may find several hydrogen bond partners in E1. The first binding mode is shown to interact with residues Ser309, Ser310, Asp311, Ala357, Val386, and Tyr388, establishing hydrogen bonds. Residues Pro381, Asp383, His384, Val385, Pro389, and Ala390 also perform hydrophobic contacts with EGCG. The second binding mode shows hydrogen bond partners with Tyr137, Thr139, Asp329, and Ser343 residues. The complex may be further stabilized by hydrophobic interactions performed with Leu27, Gly138, Thr288, Asp292, and Arg327.

**Figure 7:**
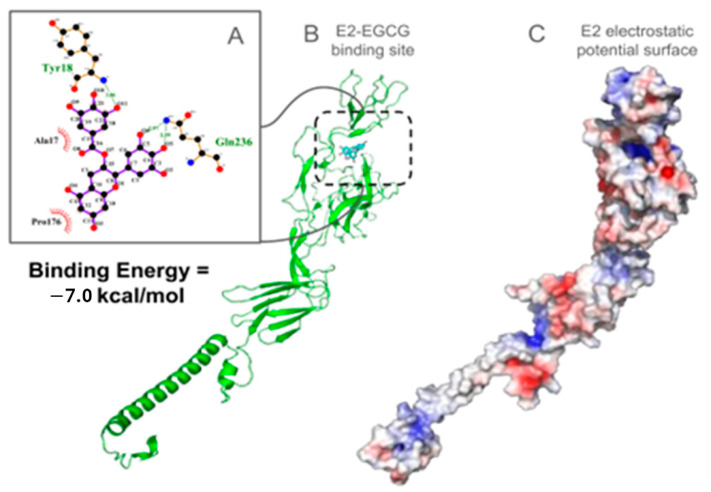
E2 protein from MAYV docked with EGCG. The best-ranked binding poses between E2 and EGCG are shown (**A**). The binding mode shows the propensity to establish hydrogen bonds with Ala17, Tyr18, Pro176, and Gln236. EGCG-docked poses on E2 protein are shown in (**B**), while the electrostatic potential surface reveals the positively charged character of the putative binding site (**C**).

The E2 protein structure from MAYV was obtained from the same PDB with the chain identifier C. As mentioned earlier, it forms heterodimers with the E1 protein, and the trimers of these heterodimers make up the virus spike. From docking simulations, two binding sites were identified for the 20 lowest energy poses. In 18 poses, the most prominent binding site is located between the A and B subdomains (see Figure 7), while the remaining two poses bind between the C and D subdomains. Both locations have significant polar contacts in the E1-E2 complex interface.

The first described binding site, between the A and B subdomains, contains residues capable of establishing hydrogen bonds with EGCG, Pro14, Val16, Ala17, Tyr18, His29, Gly72, His73, Arg123, Asp174, Pro176, Asp177, Phe241, and Gln236. Among these possibilities, Gln236 is the most frequently observed in the poses resulting from molecular docking. Upon calculating the protein electrostatic surface, a positively charged region is evident, aligning with expectations for an essential site for EGCG binding.

Despite Gln236 being the most prevalent residue in the docking assays, it does not feature in the list of crucial residues identified through virtual alanine scanning conducted by Rosetta. Conversely, Tyr18 and His29 are the residues contributing the highest energy in the E1–E2 interface, estimated to increase the binding free energy by 1.11 and 0.83 kcal/mol, respectively.

In the context of the second binding site, situated between subdomains C and D, while only two poses were identified through docking, it is noteworthy that this site includes the Arg297 residue. This residue emerges as the most crucial hot spot in the E1–E2 complex, contributing significantly to the complex free energy with a value of 3.31 kcal/mol. Both poses within this binding site exhibit the formation of hydrogen bonds with Arg297. Additionally, the protein surface electrostatic potential in this region is positively charged. Consequently, it is imperative to acknowledge this as a potential binding site for EGCG.

## 4. Discussion

MAYV is an Alphavirus emerging from the New World transmitted through mosquitoes of the genus *Haemagogus* in the tropical forests of Central and South American countries [13,48]. However, some evidence revealed that the *Aedes aegypti* vector can transmit this virus, contributing to the classification of MAYV as emerging and with the potential to establish itself in the urban cycle [7,8,48,49]. Currently, there are no specific treatments for the disease, and medications are used only to treat secondary symptoms, in addition to eliminating vector mosquitoes in endemic areas to avoid contracting the disease. Therefore, there is an urgent need to seek new antivirals capable of acting directly on MAYV [50].

In evaluating the cytotoxicity of EGCG, cell viability above 80% was observed at concentrations below 25 μg/mL. We used EGCG concentrations ranging from 6.25 to 200 μg/mL in MTT assays, and only the highest concentrations (100 and 200 μg/mL) reduced cell viability in both lines. Although EGCG is a natural compound, studies showed cytotoxicity in higher concentrations when incubated in cell lines corroborating our results [51]. A study evaluated the antiviral activity of EGCG against CHIKV, using human bone osteosarcoma epithelial cells (U2OS), demonstrating that there was low cellular damage when using a dose of 20 μg/mL of EGCG, and cell viability remained above 90% [32]. In 2017, Carneiro and collaborators also tested the cytotoxicity of EGCG using Vero E6 cells at concentrations of 0 to 200 μM, noticing a reduction in cell viability only at 200 μM [34], corroborating our EGCG cytotoxicity results in BHK-21 and Vero E6 cells.

By screening compounds already described in the literature acting against other microorganisms or other diseases, such as EGCG, it is possible to identify a broad spectrum of antiviral substances. Furthermore, we currently have a vast technological apparatus that makes it possible to screen natural products and analyze the structural diversity of these compounds. We subjected EGCG to an initial screening to evaluate antiviral activity against MAYV in vitro, showing a significant percentage of inhibition of the complete MAYV replication cycle in the MNTC tested. We used a concentration of 25 μg/mL in both cell lines, showing inhibition percentages of 90.99% in BHK-21 cells and 88.27% in Vero E6 cells. Other studies have also demonstrated the antiviral activity of EGCG, as Weber and collaborators, evaluated its effect against infection by CHIKV, a virus of the *Alphavirus* genus, in HEK 293T cells. They showed an inhibition of 60% in the CHIKV replication cycle using 10 μg/mL of EGCG [33]. Vasquez and collaborators demonstrated that EGCG at 10 μM also significantly reduced the yield of West Nile virus in Vero CCL-81 cells [35].

It was possible to observe a dose-dependent effect of EGCG in the inhibition of MAYV, as there was an increase in viral inhibition as the concentration of EGCG increased in both cell lines. Another study testing the same compound against CHIKV showed that EGCG could significantly reduce, in a dose-dependent manner, viral RNA, progeny yield, and CPE of CHIKV [32]. Weber et al. demonstrated the activity of anti-CHIKV of the EGCG, testing lentiviral vectors pseudotyped with the CHIKV envelope protein in the presence of EGCG showed significant dose-dependent inhibition of entry [33]. Raekiansyah et al. performed a focus reduction assay using EGCG against DENV and observed a decrease in infected cell foci with increasing compound doses. In addition, immunofluorescence and ELISA assays showed a reduction in NS1 protein and antigen levels with increasing compound concentration [52]. Ferraz and colleagues, in their study, used proanthocyanidin (−)-epicatechin-(4β→8) -(−)-4′-methylepigallocatechin (PAC) against MAYV. PAC showed a dose-dependent effect since reducing the concentration decreased the protection of cells against viral infection [53]. The dose–response curve showed information about the safety margin of the compound through the calculation that determines the effective concentration of 50% (EC 50), the cytotoxicity concentration of 50% (CC 50), and the selectivity index (SI). Therefore, the higher the SI value, the greater the reliability and safety of the compound [54]. EGCG presented SI = 13.26 in BHK-21 cells and 5.85 in Vero E6 cells. We obtained different values in the cell lines used, corroborating other authors who also observed this difference according to the cell line used [55]. It is worth highlighting that modest SI values in vitro assays may not present adverse effects in vivo and should be further evaluated.

When evaluating the results of the virucidal assay, we saw that EGCG acted to prevent virus replication only in BHK-21 cells, which did not occur in Vero E6 cells. These data alone argue for the possibility of modest damage to the viral particle induced by the compound. Carneiro and colleagues showed direct action of EGCG on ZIKV when pre-incubated for 1 h with 200 µM EGCG, reducing at least 1 log (>90%) in the number of foci observed [34]. Another study also showed the virucidal effect of EGCG against three clinically relevant Orthoflaviviruses (DENV-2, ZIKV, and West Nile virus—WNV). In the case of WNV, different compounds were used, and only polyphenols containing hydroxyl in R5′, such as EGCG, could inhibit it [35]. The same occurred for the hepatitis C virus, where the virions were incubated with EGCG at 25 and 80 μM, showing an inhibition rate of 95.8% and 91.6, respectively [56]. Colpittis and colleagues conducted a study with several viruses, including the Sinbdis virus (SINV), which is an *Alphavirus*, to identify the mechanism of action of the broad virucidal activity of EGCG [57]. EGCG acted on the viral particles of most of the viruses tested, including SINV. Regarding the possible mechanisms of action, the study indicated that EGCG and its products do not affect the viral envelope or influence the membrane fluidity, a characteristic of the infectivity of the particle [57]. On the contrary, they showed that EGCG interacts with tryptophan residues of the proteins of the viral particles and inhibits the binding of virions to cells. EGCG also acts in the primary binding of virions to heparan sulfate or sialic acid [57]. Therefore, it seems reasonable to assume that the mechanisms of action may be similar to this study.

In the time-of-addition assay, EGCG showed promising results, as it is a compound that presents a broad spectrum of activity, showing action on different targets. The MAYV has a complete replication cycle lasting approximately 6 h [58]. The entire process begins with the virus binding to the cell, with viral penetration between 15 and 30 min post-infection. When evaluating the effect of EGCG on this stage, we saw that it was able to inhibit the entry of the virus only into BHK-21 cells, as occurred in the virucidal assay, strengthening the hypothesis of greater affinity with receptors from different cell lines [59]. Weber and collaborators also showed that EGCG acts on CHIKV entry. For this purpose, HEK 293T cells were transduced with lentiviral vectors pseudotyped with CHIKV envelope protein. The results showed apparent inhibition at high doses of EGCG (8.3 and 25 µg/mL). Analyzing the influence of EGCG on cell binding, a reduction in the number of viral particles was noted, but without blocking them completely [33]. Another study using EGCG against CHIKV also showed an effect on entry [32]. Some studies show that EGCG acts on the entry of the hepatitis C virus (HCV), and the hypothesis is that EGCG impairs the virus’s binding to the cell surface [60,61]. Some authors suggest that catechins can inhibit or increase endosomal acidification, and since pH control is important for particle release into the cell cytoplasm, this is a hypothesis for the apparent inhibition of MAYV in this study [62,63].

After pre-treating the cells with EGCG before infection, we obtained a percentage of viral inhibition of 56.64% (*p* < 0.0001) in BHK-21 and 60.68% (*p* < 0.0001) in Vero E6, showing that the EGCG may be acting protectively in the cell lines tested. Some studies suggest that EGCG may act by blocking cellular receptors, preventing the virus from binding to the target cell. A study reported that pretreatment of MDCK cells with green-tea extracts inhibited the acidification of the endosome, inhibiting the *Influenza* virus [63]. Zhao and collaborators also demonstrated the protective effect of EGCG and EGCG palmitate on MARC-145 cells against porcine reproductive and respiratory syndrome virus (PRRSV) infection [64]. Another study involving HIV showed that EGCG binds to the CD4 receptor on T lymphocytes, preventing virus-target cell binding [65].

Evaluating the post-entry steps, we obtained a greater inhibitory effect on BHK-21 cells (85.46%) when compared to Vero E6 (57.7%) at 1 h.p.i., showing a possible impact on the internalization stage of particles in cellular vesicles. At 2 h.p.i., BHK-21 cells also obtained a greater inhibitory effect (73.20%) compared to Vero E6 (59.7%), showing an effect on the viral uncoating process and initiation of viral RNA replication. For the late stages (4, 6, and 8 h.p.i.), the same pattern was repeated, with higher inhibition values in BHK-21, demonstrating a possible effect on the assembly and release stages of the viral particle. One study testing EGCG against CHIKV also observed a significant reduction in RNA levels after viral entry at concentrations of 10 and 20 μg/mL [32]. Another study performed an immunofluorescence assay and showed that EGCG significantly decreased the level of dengue structural protein 1 (NS1) at a concentration of 100 µM [52]. The dengue NS1 protein plays a role in viral RNA replication, virion production, immune evasion, and multiple aspects of pathogenesis [66]. It also potently inhibited the expression of nonstructural protein 3 (NS3) and 5B (NS5B) of HCV. NS3 is important in transcription and polyprotein hydrolysis, and NS5B modulates viral RNA polymerization [67]. In addition, it showed inhibitory activity against Mpro (Nsp5) of SARS-CoV-2, which is responsible for the proteolysis of the mature coronavirus [68]. Therefore, since EGCG acts on enzymes in the post-entry stages of various types of viruses, it is likely that it also acts similarly against MAYV.

Western blotting analysis corroborates our previous results, showing a reduction in the expression level of MAYV E1 and NsP1 protein in EGCG-treated cells. The synthesis of the structural protein E1 in the BHK-21 cell line was impaired by EGCG treatment. This shows a possible effect in the early stages of infection, and on post-entry stages, as was noted in the time-of-addition assays. The E1 protein is a class II viral fusion protein responsible for the fusion of the viral envelope with the host cell endosomal membrane [69]. The nonstructural protein nsP1 has two types of enzymatic activity (7-guanine methyltransferase and guanyltransferase), both necessary for the methylation of the ends of the genome and subgenomic RNA during transcription. Also, it is important for the initiation of complementary RNA synthesis [69].

In this study, we also screened through an in silico analysis of the protein–ligand interactions of the MAYV structural proteins E1, E2, and C with the compound EGCG. The main reason was to find potential candidates for EGCG binding sites that would disturb the macromolecular complexes of the aforementioned proteins. Different current studies use molecular docking as a screening strategy for antiviral candidates. In our results, we show the formation of hydrogen bonds between EGCG and the Capsid protein at residues Lys130, Asp245, and Met246 and that these residues establish contact with the E2 protein of the viral envelope. Thus, the described binding site is potentially important for drugs to impair virus assembly. Furthermore, the complex is maintained with other hydrophobic contacts with residues Val127, Met132, Trp242, Asn243, Val247, Cys161, and Leu159. EGCG was also able to interact and establish hydrogen bonds and hydrophobic interactions with the E1 and E2 proteins, which are the proteins that make up the MAYV spike and are organized as trimers of the E1 and E2 heterodimers. Trimers of the E1 and E2 heterodimers make up the spikes on the viral surface that extend through the envelope bilayer and interact with the nucleocapsid C proteins. These glycoproteins control the process of clathrin-mediated viral entry into cells. They are involved in binding to cellular receptors, cellular internalization, and fusion of the viral and endosomal membranes. Therefore, the binding of EGCG to these proteins suggests that the compound may be interfering with the initial processes of the viral replication cycle [3].

Therefore, this work was the first to demonstrate the effectiveness of EGCG against the Mayaro virus using different cell lines. EGCG affected various stages of the MAYV replicative cycle, virucidal effect, and protective effect on cells. Our results suggest that EGCG is a strong candidate as a broad-spectrum antiviral, as it already acts against other Alphaviruses. This broad effect is excellent considering the epidemiological situation of countries affected by arboviruses, in which viruses co-circulate, making diagnosis difficult and involving thousands of people.

## Data Availability

The original contributions presented in this study are included in the article/Supplementary Material. Further inquiries can be directed to the corresponding author.

## Supplementary Materials

The following supporting information can be downloaded at https://www.mdpi.com/article/10.3390/v17020258/s1, Figure S1: Cytotoxic effect of compounds. BHK-21 and VERO E6 cells were treated with the compounds at different concentrations for 24 h, and cell viability was subsequently analyzed using the MTT assay. The black line shows the cytotoxicity threshold of 80%.VC: vehicle control: DMSO. Values from three independent events carried out in triplicate, with standard deviation (SD); Figure S2: E1 protein from MAYV docked with EGCG. The two best-ranked binding poses between E1 and EGCG are shown. The first binding mode shows the propensity to establish hydrogen bonds with Ser309, Ser310, Asp311, Ala357, Val386, and Tyr388 (**A**). The second binding mode shows interaction by hydrogen bonding with Tyr137, Thr139, Asp329, and Ser343 (**B**). EGCG-docked poses on E1 protein are shown in (**C**), and the electrostatic potential surface reveals the negatively charged character of the putative binding sites (**D**).
