# Supplementary material for: In Vitro Evaluation of the Antiviral Activity of Polyphenol (-)-Epigallocatechin-3-Gallate (EGCG) Against Mayaro Virus
Source: Viruses. 2025 Feb 14;17(2):258. doi: 10.3390/v17020258 (PMC11860591; doi:10.3390/v17020258)
Supplement: Supplementary file 1 [file viruses-17-00258-s001.zip › viruses-3405748-supplementary.pdf]

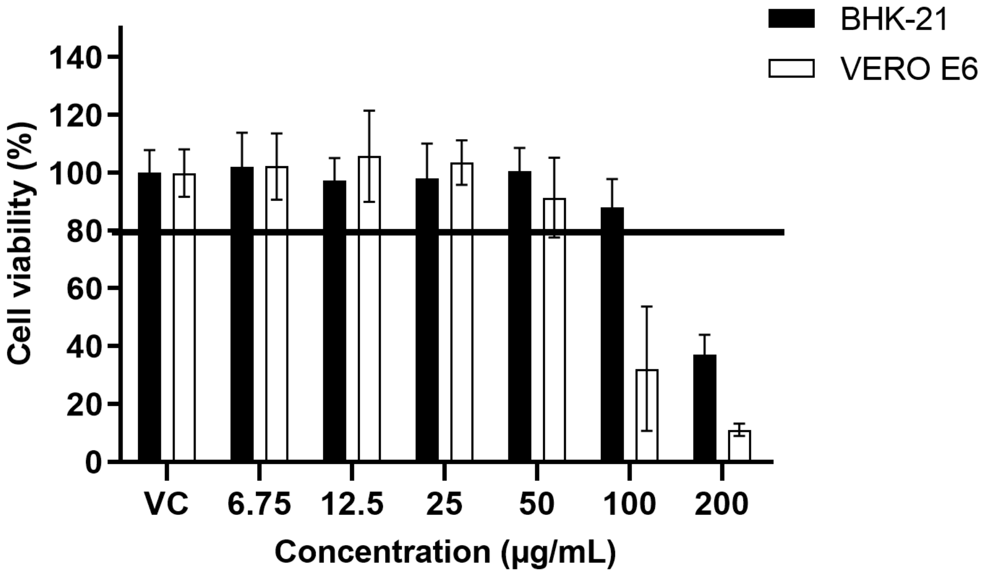

Figure S1. Cytotoxic effect of compounds. BHK-21 and VERO E6 cells were treated with the compounds at different concentrations for 24 h, and cell viability was subsequently analyzed using the MTT assay. The black line shows the cytotoxicity threshold of 80%.VC: vehicle control: DMSO. Values from three independent events carried out in triplicate, with standard deviation (SD).

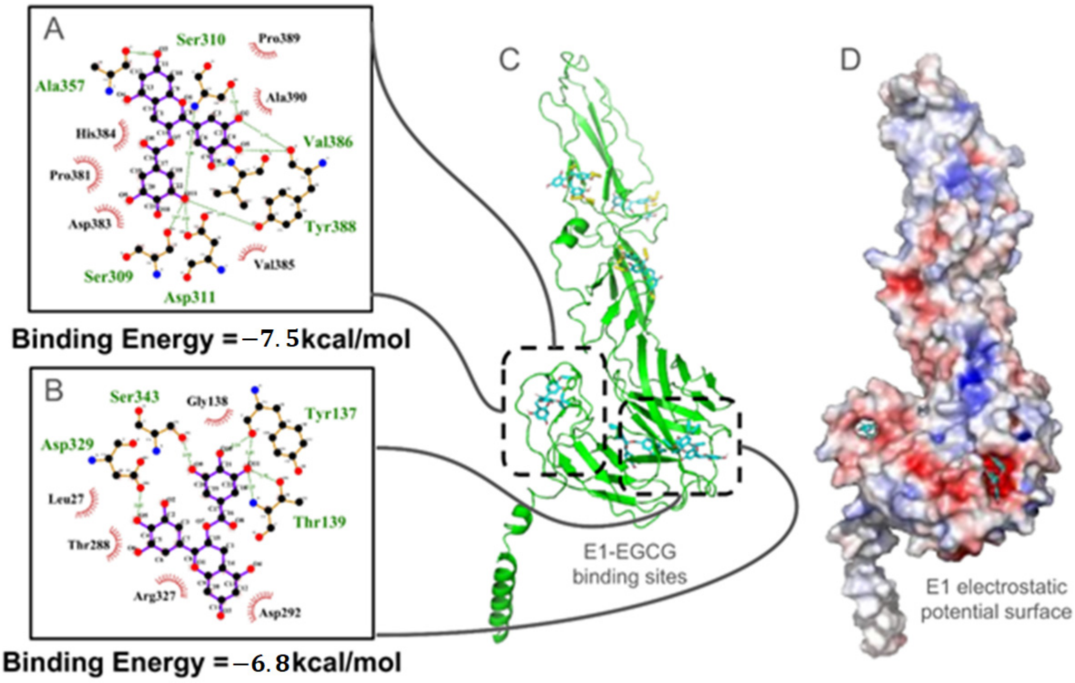

Figure S2. E1 protein from MAYV docked with EGCG. The two best-ranked binding poses between E1 and EGCG are shown. The first binding mode shows the propensity to establish hydrogen bonds with Ser309, Ser310, Asp311, Ala357, Val386, and Tyr388 (**A**). The second binding mode shows interaction by

hydrogen bonding with Tyr137, Thr139, Asp329, and Ser343 (**B**). EGCG-docked poses on E1 protein are shown in (**C**), and the electrostatic potential surface reveals the negatively charged character of the putative binding sites (**D**).
